# Electroencephalogram-based decoding cognitive states using convolutional neural network and likelihood ratio based score fusion

**DOI:** 10.1371/journal.pone.0178410

**Published:** 2017-05-30

**Authors:** Raheel Zafar, Sarat C. Dass, Aamir Saeed Malik

**Affiliations:** Centre for Intelligent Signal and Imaging Research (CISIR), Universiti Teknologi PETRONAS, Bandar Seri Iskandar, Perak, Malaysia; University of Minnesota, UNITED STATES

## Abstract

Electroencephalogram (EEG)-based decoding human brain activity is challenging, owing to the low spatial resolution of EEG. However, EEG is an important technique, especially for brain–computer interface applications. In this study, a novel algorithm is proposed to decode brain activity associated with different types of images. In this hybrid algorithm, convolutional neural network is modified for the extraction of features, a t-test is used for the selection of significant features and likelihood ratio-based score fusion is used for the prediction of brain activity. The proposed algorithm takes input data from multichannel EEG time-series, which is also known as multivariate pattern analysis. Comprehensive analysis was conducted using data from 30 participants. The results from the proposed method are compared with current recognized feature extraction and classification/prediction techniques. The wavelet transform-support vector machine method is the most popular currently used feature extraction and prediction method. This method showed an accuracy of 65.7%. However, the proposed method predicts the novel data with improved accuracy of 79.9%. In conclusion, the proposed algorithm outperformed the current feature extraction and prediction method.

## Introduction

Decoding brain activity involves the reconstruction of stimuli or brain state from the information measured using different modalities like electroencephalogram (EEG) or functional magnetic resonance imaging (fMRI). Stimulus or brain state information is encoded in the brain and is present in the form of neuronal activity. Decoding this recorded neural information and associated changes in brain activity can be used to predict the specific tasks or stimuli that caused the response in the brain. Different neuroimaging techniques can be used to find differences in brain activity during different tasks or conditions. For example, in fMRI, the measured signal is the blood-oxygen-level dependent signal. In contrast, EEG records electrical signals that indicate activity and responses in the brain.

EEG is a widely used technique that can measure the changes in electrical voltage on the scalp, induced by cortical activity [1]. In EEG, data is collected from multiple channels (EEG electrodes) that record the signals corresponding to the activity in different cortical regions. EEG time-based and frequency-based features are extracted from a continuous time series and supervised learning algorithms have been applied to find the discriminative features between the states or stimuli. [2, 3]. However, the EEG signal is buried under noise, increasing the difficulty in decoding brain activity. Moreover, decoding the neuronal activity is also dependent upon the performance of machine learning algorithms used, which include support vector machines (SVM) and logistic regression (LR) [4].

In a pioneer study, Haxby et al. [5] distinguished brain activity patterns for images of different categories such as faces, houses, animals, chairs and tools using fMRI. Recent research in brain decoding still has focused on fMRI for data collection [6], [7], [8], [9], [10], [11, 12], [13] and [14]. However, EEG is well-established, for use in brain–computer interface (BCI) applications [15–19], epilepsy [20] and seizure detection [21]. It is also used for the application of controlling robotics [22]. Due to low spatial resolution of EEG, only few studies are available in brain decoding. While, in recent years, the spatial resolution has improved with 256- and 512-channel EEG caps, which provides a more detailed data with improved accuracy. A study by Cruse et al. [23] monitored the consciousness of vegetative patients using EEG and compared the obtained results with an existing fMRI results. The comparison showed that EEG had better accuracy in detection of patients’ response to commands than fMRI. In the experiment, the researchers checked the consciousness of the vegetative patients using EEG and found better results compared with fMRI; however both of these studies were done separately. Taghizadeh-Sarabi et al. [24] achieved 70% overall decoding accuracy in an experiment using 19 channel EEG equipment. In another study, Douglas et al. [25] stated that EEG data can outperform fMRI data in decoding the belief decision. Moreover, decoding the taste categories was recently performed by Crouzet et al. [26] using EEG. The above mentioned studies suggest the potential of EEG for decoding brain activity. However, more research is required to increase the understanding of brain using EEG.

In this study, Convolutional neural network (ConvNet) is used to decode brain activity patterns. ConvNet belongs to a broader family of machine learning methods; it is based on learning from the representation of data. The primary advantage of ConvNet is the replacement of handcraft features with automatically derived features found in efficient algorithms. In contrast with neural networks, deep neural networks have more than two hidden layers [27]. In recent years, ConvNet has achieved great success in different applications for recognition tasks. These applications include video, images, text and speech [28–32]. ConvNet is a most popular architecture of deep learning. ConvNet works better with images and video data compared with different existing hand-crafted feature extraction methods. [33], and it has also performed well in many other applications involving handwriting, speech recognition [29] and video classification [34].

ConvNet is a complete framework consisting of a convolutional layer, pooling layer and fully connected layer which is used as a classifier. In other words, ConvNet is a structure which takes raw data as input and gives the final classification/prediction results. There are many advantages related to ConvNet which are detailed in the literature. The primary attribute of interest in ConvNet is that it can directly classify the raw signal and can integrate signal processing functions. It is easy to extract features in ConvNet because there is no need to know about the type of the features. ConvNet extracts the most discriminative features by constructing high level features over the whole data set. ConvNet can also be trained easily using traditional methods due to its constrained architecture, which is specific to input for which discrete convolution is defined, such as signals and images. Due to all these advantages, especially easy implementation and good accuracy, ConvNet is currently an active research area.

ConvNet is successfully being used in many applications and it has also worked well with moderate data sets both in EEG and fMRI [35–38] [39]. Cecotti et al. [40] used ConvNet for P300 detection in a BCI application and obtained a high degree of accuracy. ConvNet can also handle the variations present in EEG signals [41]. Plis et al. (2014) [35] showed that, by using different layers of deep belief network, accuracy can be increased compared to other classifiers. ConvNet has also been used to extract features from EEG time series [36, 37, 42]. These studies have demonstrated potential benefits of the use of ConvNet in neuroimaging, even with moderately sized data sets. ConvNet is the most popular method for all applications including image and speech recognition. However, in the neuroimaging domain, it is relatively unexplored due to some shortcomings. It has high computational cost and requires too much training data, which is generally impossible in brain studies. Second, no well-established model of ConvNet exists for brain studies [43]. Machine learning algorithms have become very popular in recent years for training classifiers for decoding/prediction of stimuli, behaviors, mental states and other purposes [44]. Since, machine learning algorithms have a key role in the process of decoding; choosing the correct classifier and its parameters is very important for performance. Support vector machine (SVM) is the most popular and widely used machine learning approach. This approach is normally based on supervised learning algorithms [45]. Score fusion techniques can be another way to predict brain activity efficiently and are being used in biometric systems parallel to SVM [46, 47].

Decoding the human brain is quite mature in case of fMRI and many studies were presented in last 15 years [5–14]. These studies used different fMRI scanners (3T-7T), models, experiment design and number of subjects. These studies successfully decoded the human brain activity with good accuracy, starting from simple visual task to complex natural images and even with movies [48]. Currently fMRI has shown best results with available hardware. Functional MRI measures the neural activities indirectly and the data is normally taken after every 2 sec which means the temporal resolution of fMRI is limited. This restriction may be overcome in the future with better machine for the collection of data. In case of EEG, this research area is still progressing and only few studies have been reported in past years [24, 26] which shows lack of research in this area. The same is stated by Agrawal et al., in an article “EEG and NIRS offer portable solutions but with their signal quality no substantial results for predicting brain activity have been reported” [49].

In this study, the purpose is to investigate the brain states using EEG. EEG is a portable and cost effective solution to fMRI; if better or comparable results are achieved, it would be more helpful in the research especially in medical field. It is easy to collect EEG data especially in case of patients and neural activity can be detected promptly due to its high temporal resolution. Functional MRI is widely used in research and is considered to be a better modality for brain decoding, as it can extract more information from specific regions of the brain compared to EEG. In EEG, the quality of data is considered to be vulnerable compared to the quality of fMRI data so it is difficult to extract more information from a specific brain region using EEG [26]. Hence EEG is not a popular modality to decode brain activity from specific brain regions. Some new studies have used multivariate pattern analysis (MVPA) to decode data on brain activity which are acquired from the whole brain instead of from a particular brain region [11, 26, 50–52]. For decoding the brain activity patterns, MVPA is an emerging technique and has proven as a highly useful technique for decoding of different patterns of brain activity [11, 53, 54]. This new concept of MVPA and more EEG channel device encourages neuroscientists to decode brain activity using EEG, as EEG with a higher number of channels, also improves spatial resolution. This is the reason we proposed an algorithm to improve the decoding accuracy with EEG data which has new machine learning technique (ConvNet) and a different prediction method likelihood ratio based score fusion (LRBSF) in neuroscience.

The motivation for this study is to propose a hybrid algorithm. The proposed algorithm is hybrid, which consists of different existing and proposed approaches in neuroscience including data representation, CNN for feature extraction, t-test for feature selection and LRBSF for prediction. The main focus is on three main aspects. First, decoding brain activity using EEG data, which has been done in only a few previous studies. Second, modification of ConvNet architecture according to a 1D EEG signal and use of the architecture with limited brain data. The limited brain data consists of approximately 50 trials per category, which is quite less than image recognition data sets such as the Mixed National Institute of Standards and Technology (MNIST) data set which has a training set of 60,000 examples [55]. Last, the use of LRBSF instead of SVM for prediction and validation of results by comparing the results with SVM. In short, the main contribution/novelty of this study is an algorithm which consists of ConvNet architecture and LRBSF along with conventional method (t-test) used for brain studies. In addition, the filters used in ConvNet architecture are also designed according to EEG data, moreover EEG data is arranged in such a way that instead of averaging the data, the data of every image becomes the part of the final analysis directly. To validate the proposed algorithm it is compared with widely used existing methods.

## Material and methods

### Participant information

Data were taken from 30 participants; however data from only 26 participants were used for the final analysis after application of exclusion criteria. Data from two participants were excluded due to the presence of a large number of artifacts, while two other participants showed low accuracy during the initial analysis of baseline and the task. All participants submitted the written consent form before the start of the experiment. The age of all participants was between 24 and 34 years and the mean age was 30 years. The study protocol was approved by the Universiti Teknologi PETRONAS (UTP) ethics committee under UTP Reg. No: 13–10 and the EEG data was recorded at UTP.

### Stimuli

A total of 260 grayscale photographs were presented in a single session (21 minutes). All images were taken from the internet, freely available and had already been used in a previous study [8]. Every image was of size 500*500 pixels with a 4*4 pixel fixation spot in the middle of every image. All images were masked with a circle (20^o^ diameter) and had the same contrast and brightness.

### Experimental procedure

**Fig 1:**
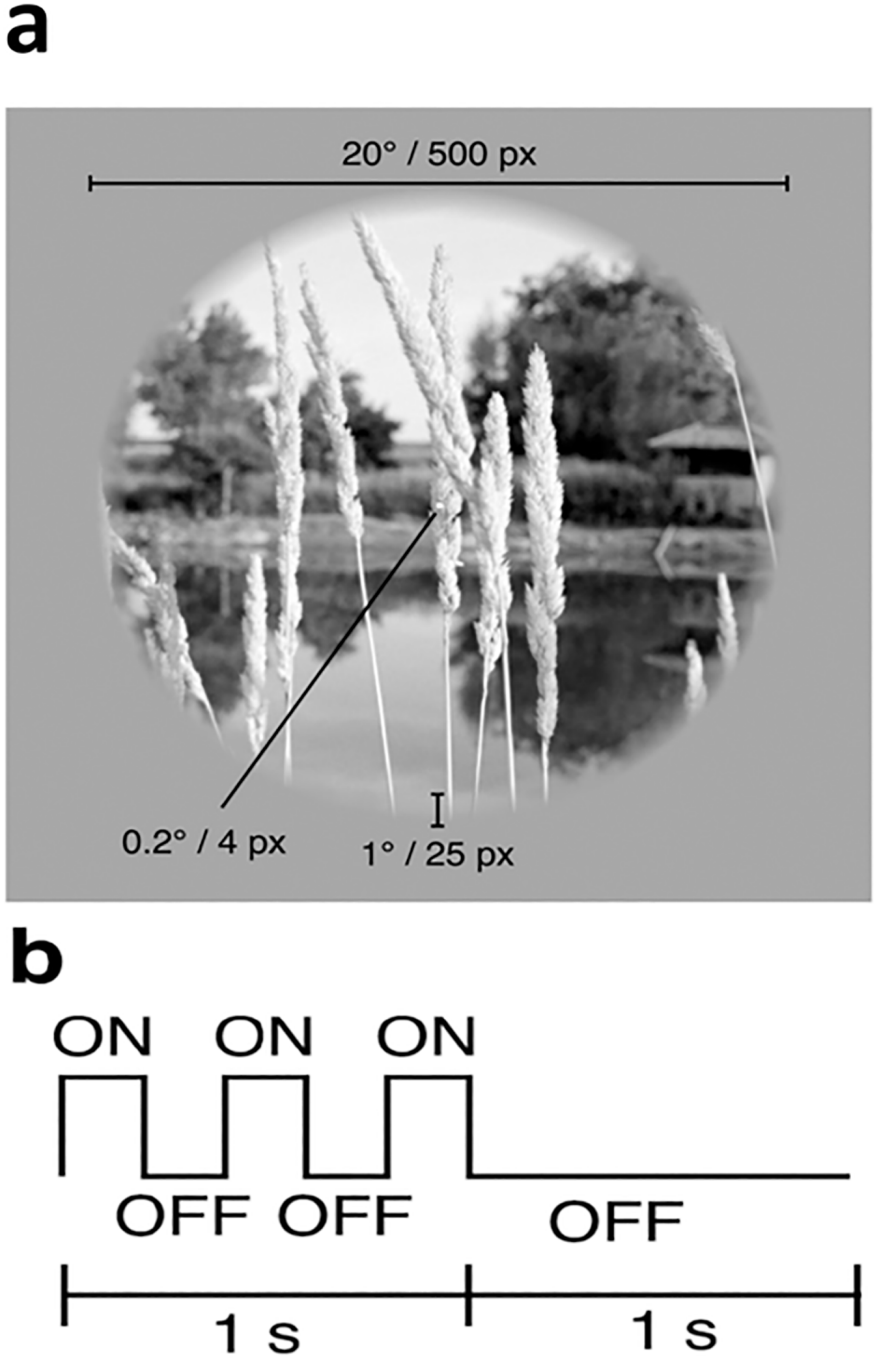
Experimental paradigm: Images of five categories (humans; animals; buildings; natural scenes; fruits) were presented for 1sec with a grey background and rest period of 1 sec. The stimuli consisted of sequences of grayscale natural photos. a) Spatial characteristics. The photos were masked with a circle (20° diameter) and placed on a gray background. The outer edge of each photo (1° width) was linearly blended into the background. A central white square (0.2° side length) served as the fixation point. b) Temporal characteristics. The photos were presented for 1sec with a delay of 1 sec between successive photos. Each 1-s presentation consisted of a photo being flashed ON-OFF-ON-OFF-ON where ON corresponds to presentation of the photo for 200 ms and OFF corresponds to presentation of the gray background for 200 ms [8].

The images were divided into five categories: human, animal, building, natural scenes and fruits. Every stimulus (grayscale photograph) appeared on the screen for one second with 200 ms on and off, and with a rest period of one second after every stimulus. All images appeared twice on the screen. Participants were instructed to focus on the screen and try to recognize the category of the image. This was a non-response task. The participants were told that they should only view and think about the category. The complete experiment paradigm is shown in Fig 1.

### EEG data collection

Continuous EEG data were recorded with a 128 channel Electrical Geodesics Incorporated (EGI, Eugene, OR, USA) system with a sampling frequency of 250 Hz. Cz was the reference electrode for all other electrodes. The raw EEG data were amplified with the EGI NetAmps 300 amplifiers with bandpass filter set from 0.1 to 100 Hz. The threshold for impedance was 50 KΩ. Net Station 5 Software [56] was used to record, save and display the online recordings of EEG data.

### Pre-processing of EEG data

The data were exported to Brain Electrical Source Analysis (BESA, Gräfelfing, Germany) software for pre-processing where the signals were filtered from 0.3 to 30 Hz using a bandpass filter. This was done to remove DC components and muscular artifacts which have high frequencies; moreover, these were the most relevant frequencies during a visual task [57]. Eye blink (EOG) artifacts were corrected using an adaptive artifact correction method present in BESA. All data were visualized manually and any unwanted signals were corrected manually. In BESA, we removed any type of artifact by selecting a default block epoch and removing the unwanted patterns. The default block epoch is first need to define and will later be used for the pattern search. In this way artifact is detected by defining a block with in a particular period of time only, which can later be used to search the same artifact pattern from the whole data set. After removing artifacts, the file was exported to MATLAB (MathWorks, Natick, MA, USA) for further analysis. Data from every trial were separated in MATLAB and no single trial or image was excluded from the analysis.

### Proposed method

**Fig 2:**
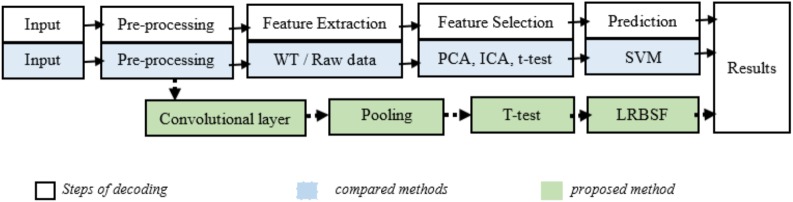
Block diagram of proposed and compared methods for brain decoding. The proposed method is shown in the green boxes while compared methods are shown in blue boxes.

The analysis of EEG data consisted of the following steps: feature extraction, selection of the best informative features and classification/prediction. The proposed algorithm/framework consisted of the following different stages. EEG data were arranged in such a way that every image became part of the final analysis directly. Features were extracted and enhanced using ConvNet and the extracted EEG features were arranged in a row vector as is normally done in fMRI analysis. Significant features were found statistically with a t-test and prediction was done using a method novel in brain studies (LRBSF). Fig 2 explains the details of the proposed method with complete steps for decoding along with the steps of compared methods. The main contribution in this hybrid algorithm was the modification of the basic ConvNet model (LeNet) for extraction of features from EEG data, the different arrangement of 128 channels of EEG data and LRBSF for prediction.

#### Neural network architecture

Network topology is important in ConvNet. ConvNet has three main layers: a convolutional layer, a pooling layer and a fully connected layer. In our proposed algorithm, only two layers (one convolutional and one pooling layer) with several maps were used. In this algorithm, the filters are vectors instead of matrices because the data are 1D. Matrices are generally used in image recognition [40]. Different sizes of 1-D filters are used along with large and small pooling layers; the best combination is reported in this study. The idea of enhancing the features is to extract detailed information from different activity patterns generated in association with the specific stimulus. Finally, the extended features are reduced using a feature selection method. The dimension of convolutional and pooling layers are dependent on the size of relevant filters, however there is no specific rule defined to select the size of filters and number of ConvNet layers. This is mostly dependent on the model which is being used. These models are mostly fixed with number of layers and size of input data. For example the AlexNet came in 2012, and used 11*11 size filter at the first layer while ZF Net came in 2013 with input image size of 224*224 (quite large) and 7*7 filter at first layer. This means the models are mostly designed for specific applications. In different ConvNet architectures, the number of layers are also different, for example GoogLeNet (2015) is considered to be a one of the best model with 22 layers [58]. However, unfortunately in neuroscience there is no such model exists since all the current models are designed with engineering goals and not for brain computations [43]. One of the main reasons is that the number of samples are limited in most of the neuroimaging datasets [59].

#### Learning

**Fig 3:**
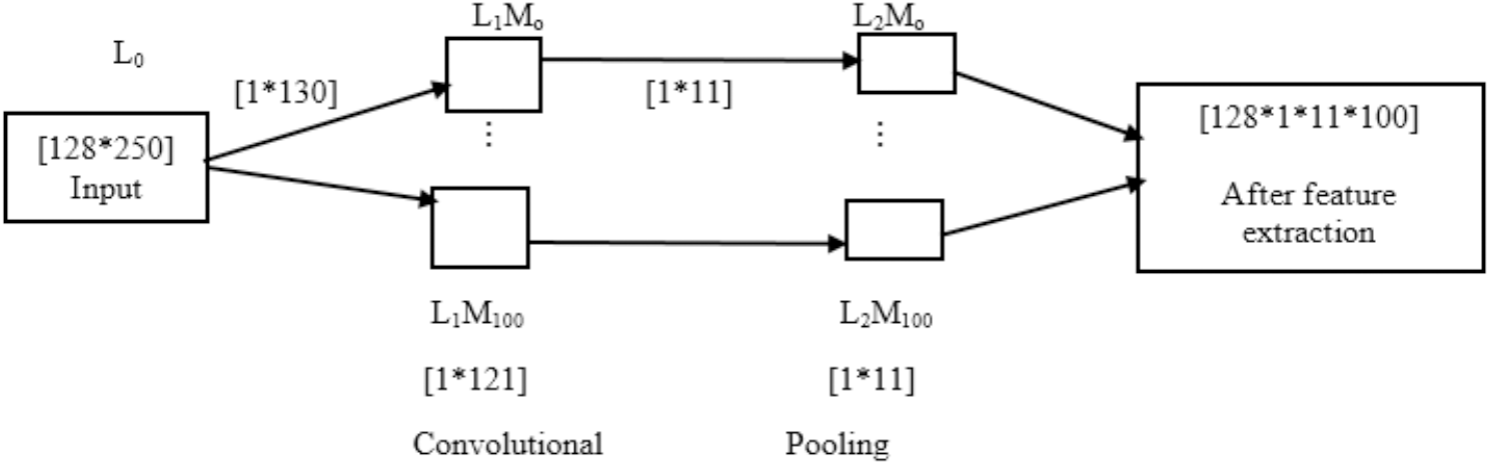
Schematic of the deep neural network where L1 and L2 are the layers and M defines the maps of convolutional layer.

In this network, there was an initial input matrix of (128 × 250) for every stimulus, in which the number of channels was arranged in the rows and number of samples in columns as shown in Fig 3. If N_e_ is the number of electrodes and N_s_ is the number of samples, then the input layer could be defined as 0 ≤ *i* ≤ *N*_*e*_ *and* 0 ≤ *j* ≤ *N*_*s*_.

Let the neurons in this network be defined by *f*(*l*, *m*, *p*), where *l*, *m*, and *p* are the layer, map and the position of neurons in the map, respectively. If there is only one map in the layer, then the value of a neuron is $y_{m}^{l}(p)=y^{l}(p)$. The general equation is

$$y_{m}^{l}(p)=g(\sigma_{m}^{l}(p))$$ (1)

Where *g* depends on the layer and is known as a sigmoid function. This sigmoid function is approximately linear and its value lies between 0 and 1. This function is normally used at a convolutional layer and represents the convolution of the input signal. In this study, the classical sigmoid function was used which is as follows.

$$g(\sigma )=\frac{1}{1+{exp}^{-\sigma }}$$ (2)

As discussed above, different vector filters were used, but results were reported for the filter size of (1 × 130) as we have 128 channels and 250 samples. This was the best filter size for these parameters. These filters were the neurons which had input weights and output values. In the model, each filter was replicated throughout the entire visual field. These filters shared the same weight vector and bias, which formed a feature map. The details of layers and maps of every layer are shown in Fig 3. The input neurons and the weight connections are represented by a scalar product which is denoted by $\sigma_{m}^{l}(p)$. These weight connections were between input neurons (vectors in this study) and the neurons present in the map in the layer (vectors in this study). The efficiency of learning could be increased by increasing the weight sharing, which reduces the number of free parameters being learnt so that 100 weights were defined during formation of a feature map, which is obtained as follows.

$$h_{ij}^{k}=\tanh((W^{k}*{y)}_{ij}+b_{k}$$ (3)

Where *h*^*k*^ defines the feature map at the *k*^*th*^ layer and *W*_*k*_ and *b*_*k*_ are the weights and bias of the filters.

In the next step, pooling was performed. The pooling procedure was a simple down sampling of the feature map. Pooling was done with 1*11 filter size because this was related to the size of the feature map. Finally, for every row vector, an output of (1 × 1100) was obtained which was reduced by applying feature selection techniques.

#### Feature selection

Feature selection is a method used to improve accuracy and decrease training time. There are different feature selection methods such as region of interest (ROI), principal component analysis (PCA), independent component analysis (ICA), t-test and many more. In ROI based studies, [8, 13] EEG data are restricted to a few channels for further analysis. There are some studies on EEG which have used MVPA, however in a recent study, Crouzet et al [26] showed that in EEG, MVPA performance is very good compared to individual channels.

In this study, MVPA was used along with ConvNet and the number of features was large due to the 128 channel EEG equipment. The dimensionality of the data was further increased using the convolutional layer of ConvNet. Therefore, feature selection was necessary. During feature selection, the n most significant features between the categories were extracted using a t-test as follows.

$$t^{\left(j\right)}=\frac{\mu_{A}^{\left(j\right)}-\mu_{B}^{\left(j\right)}}{\sqrt{\frac{\sigma_{A}^{\left(j\right)}}{\left|A\right|}+\frac{\sigma_{B}^{\left(j\right)}}{\left|B\right|}}}$$ (4)

Where *μ*_*A*_ and *μ*_*B*_ denote the average while *σ*_*A*_ and *σ*_*B*_ denote the variance of the data points *A* and *B* respectively and j is one of the combinations between five categories.

In the t-test, the highest t-values or lower p-values were selected, which yields the significant features in separating the classes. The features with lowest p values (p < 0.05) were considered to be significant and were ranked with lowest p values. The subsets were made from 50 to 5,000 features and the features with p > 0.05 were excluded from the analysis because they do not have much impact during analysis.

Because ConvNet is a complete framework, most of the studies have used the ConvNet classifier (softmax) for prediction/classification [37]. However, ConvNet is a complex structure, especially with the fully connected layer. Thus, some studies have shown that, in ConvNet, SVM at the top layer works better than softmax [60, 61]. In this study, prediction was done using a new method (LRBSF) instead of SVM. In LRBSF, fusion of match scores was done based on a likelihood ratio test. This method has never been used in brain decoding studies. The results are compared with the most successful, reliable and popular classifier for brain studies, SVM.

#### Likelihood ratio based score fusion

Likelihood ratio-based score fusion (LRBSF) is based on density based score fusion and can be used to directly attain optimal performance. In this procedure, a kernel density estimator (KDE) is used to estimate the densities of different classes using only training data and fusion is done between a match score and these densities. The estimation of the class of the score is done using a likelihood ratio test (LRT) [46]. Score based fusion is common in biometric systems but has never been used in prediction of brain activity.

LRT is a method for evolving a hypothesis test, in which both the alternative and null hypotheses exist. Let the null hypothesis be denoted by M_0_ and the alternative hypothesis by M_1_; f_0_(x) and f_1_(x) are the conditional joint densities of the k match scores given the null and alternative hypotheses respectively, where x = [x_1_,x_2_,x_3_,⋯,x_k_]. Let the match scores of K different given matchers be represented by X = [X_1_,X_2_,X_3_,⋯,X_k_]. The match score of the kth matcher is represented by the random variable X_k_ where k = 1,2,⋯,K. The purpose of the test is to assign the observed match score vector X to one of two classes i.e. M_0_ or M_1_. H_0_ is the null hypothesis which should be rejected and H_1_ is the alternative hypothesis. According to Neyman-Pearson theorem, [62] for testing a hypothesis H_0_: f(x) = f_0_(x) against H_1_: f(x) = f_1_(x), the likelihood ratio test which rejects H_0_ in favor of H_1_ has the form

$$\Psi (x)=\frac{f_{0}(x)}{f_{1}(x)}\leq \eta$$ (5)

P(Ψ(x)≤η)=α (6)

If Eqs 5 and 6 are satisfied for threshold η, this is the most powerful test among all level α tests.

KDE is a non-parametric way to estimate the probability density function (pdf) of a random variable. Without any presumptive distributional properties, it is used for the estimation of a distribution on a given set of data samples [63]. The advantage of non-parametric estimation is that it does not have a fixed structure and does not depend upon all the data points for estimation. KDE is widely used in many fields and applications [64, 65] [66, 67].

#### Fusion of match scores based on likelihood ratio test

As we have discussed earlier, five different categories were used in this study. One to one decoding was done between all five classes in a manner similar to the one against one method in multi class SVM, in which the process is repeated *k*(*k* − 1)/2 times and every time the decoding accuracy is found between only two conditions. After selecting the significant EEG features, the feature vector was rearranged according to highest t-values and subsets of features were made. Every time the training and testing data were separated out randomly; the probability density function of both classes was estimated using a training data set and fused with the test vector to find the prediction accuracy between two classes. The decision about the class of the test vector was done using the likelihood ratio test described in Eq 1. In the first case of our experiment, there were two conditions: human and animal. If M_1_ represented the human and M_0_ represented the animal class, then quality based likelihood should be greater than 1 for the M_1_ and less than 1 for the M_0_ class. In this technique, density based scores were fused for explicit estimation of M_1_ and M_0_ match score densities. The advantage of this approach was that it could directly attain ideal performance for any desired point and was based on the estimation accuracy of the scores. The KDE was used to estimate the densities of the classes.

#### Fusion of match scores and their performance

To define the fusion of match scores based on likelihood ratio, a vector of match scores and estimated densities was required to compute the likelihood ratio fusion. Let K is match scores vector with different number of features and ${\hat{f}}_{1}(x)$ and ${\hat{f}}_{0}(x)$ be the estimated densities of human class and animal class, respectively. The x should be assigned to the M_1_ class

$$if\;LR(x)\geq \eta ,\;\mathrm{where}\;LR(x)={\hat{f}}_{1}(x)/{\hat{f}}_{0}(x)$$ (7)

and η is the decision threshold which is found based on the overall accuracy.

The Eq 7 can be re-written as

$$\hat{f}(x)=\prod_{k=1}^{K}{\hat{f}}_{k}(x_{k})=\sum_{k=1}^{K}log{\hat{f}}_{k}(x_{k})$$ (8)

During analysis of multi-class decoding, LRBSF is applied to all five classes simultaneously by extending the two classes method. Let c_1_, c_2_, …., c_m_ are different classes then the likelihood ratio for every class is found using

$$\hat{f}(x)=\sum_{j=1}^{p}log\;{\hat{f}}_{c_{k,j}}(x)=S_{k}$$ (9)

For the right choice of the decision among all five classes, the following rule is applied, which allows a decision about the right class based on the maximum value.

$$\mathrm{Rule}:\;\mathrm{Assign}\;\mathrm{to}\;\mathrm{class}\;k_{o}\;\mathrm{iff}\;\underset{1\leq k\leq m}{\max}S_{k}=S_{k_{o}}$$ (10)

#### Significance test

In this study, a Monte-Carlo cross validation (CV) procedure was applied to check the performance of SVM and the proposed method. In Monte-Carlo CV, data are divided into k equal sized folds, which is the same as in k-fold CV. However, unlike k-fold CV, in which there is no repetition of any sample in different folds because the division of folds is sequential, in Monte-Carlo CV the division of folds is random, which means there is a chance of repetition of samples in different folds. The advantage of Monte-Carlo CV is that it can explore many possible partitions and give extra variations in the analysis which produces more reliable results. The process of Monte-Carlo CV is repeated 100 times and every time the entire data are divided randomly into a 90% training set and 10% testing set. The average performance of 100 repetitions is reported in the study.

## Results

In this section, results are reported for different methods of feature extraction and prediction/classification. However, the arrangement of EEG data and the method of feature selection is the same for all the methods. The classification accuracy of MVPA may depend on the number of selected features [6] so we chose the *n* most active features per class. The value of *n* lies between 50 and 5,000 features in discrete steps which start from 50 and finish at 5,000 with an increase of 50 features every time, so there are 100 levels of feature selection.

In all the presented results data of every image was analyzed separately. In the experiment, every image appeared twice means there were two presentations of every image.; Initially data from every presentation (trial) of every image was separated out. Every presentation of image was appeared on the screen for 1 sec followed by a rest period of 1 sec. The EEG data had sampling frequency of 250 Hz, so every presentation (trial) of image and its corresponding rest period had 250 samples. In this way every presentation of image and rest period had a matrix of size 128*250; 128 are representing the number of channels in the rows and 250 are samples in the column. For further analysis we took average data of both presentations for every image. In this way instead of single trial analysis we did analysis by taking the average of two trials which can give better quality of data. This averaging technique is used in previous fMRI studies [8, 10, 13] related to decoding the human brain. In all presented results, analysis of every image means the average of both presentations (trials) of the image.

### Decoding results between task vs baseline

Initially, a basic analysis is done in which the statistical difference of the task with respect to the baseline is found. The features are extracted from every image and compared with the corresponding baseline. These Features are extracted using a discrete wavelet transform (DWT), a t-test is applied for the selection of significant features, and SVM is used for classification. An average accuracy of 96% is achieved in this analysis with an average number of 712 features, because the results are found with a different number of features ranging from 50 to 5,000. We have also found which EEG channels out of 128 are more significant in this simple task or in other words which have more significant information for a visual task. These significant channels are mostly from the occipital and temporal regions and these channels are later used for further analysis.

### Decoding results of five classes using existing methods

In the main analysis, instead of two conditions i.e. task and baseline, the data are divided into five categories and one against one (pairwise) SVM classification is done for all conditions. In pairwise classification, *k*(*k* − 1)/2 SVMs are trained for k classes to distinguish the samples of one class from the other. Decoding accuracy is achieved by classifying the correct test vectors and a confusion matrix is made to assess the performance of the classifier.

**Fig 4:**
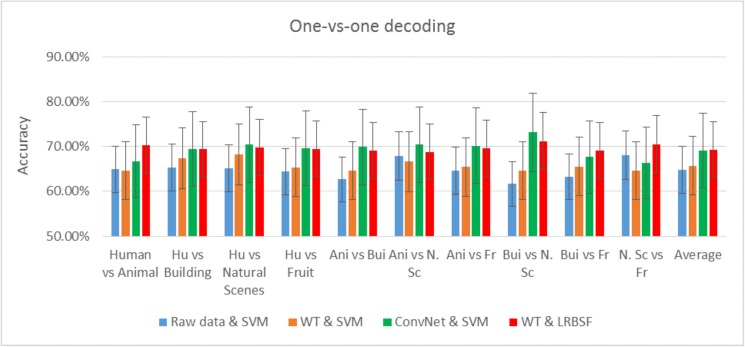
Pairwise decoding results of five classes using existing methods and combination of existing and proposed methods.

The most common and popular feature extraction and prediction/classification methods in EEG data analysis are the wavelet transform (WT) and SVM, respectively. They are implemented separately and also with a half contribution of the proposed method; decoding results for the combination of ConvNet & SVM and WT & LRBSF are also found. The average result from all participants is shown in Fig 4 along with the existing methods, which show that even a half contribution of the proposed method is better than the existing one.

### Decoding results using proposed method

In the previous section, both ConvNet and LRBSF were individually implemented with SVM and WT, respectively for five different conditions which were classified using pairwise classification. In this subsection, the results from the combined ConvNet and LRBSF method are discussed, which are far better than all previous cases. During the analysis of the proposed method, accuracy, sensitivity and specificity of all 26 participants were found using the confusion matrix and the final results were averaged across participants for each category. The average accuracy, sensitivity and specificity along with the best and worst accuracy of any participant for each category is mentioned in Table 1. These results are one-versus-one (pairwise) classification of all five categories.

**Table 1:** Performance for all categories using the proposed method (ConvNet & LRBSF). Best and worst performance of individual participant is also mentioned.

	Average of 26 participants			Individual participant	
Feature selection with two sample t-test	Accuracy	Sensitivity	Specificity	Best accuracy	Worst accuracy
**Human vs Animal**	77.1%	77.2%	76.9%	87.1%	73.1%
**Human vs Building**	79.1%	83.7%	75.4%	86.2%	72.2%
**Human vs Natural Scenes**	80.1%	79.07%	80.7%	86.3%	73.8%
**Human vs Fruit**	78.7%	75.5%	81.2%	89.1%	73.3%
**Animal vs Building**	81.5%	76.56%	88.88%	87.4%	72.5%
**Animal vs Natural Scenes**	83.1%	88.6%	76.6%	85.9%	73.6%
**Animal vs Fruit**	77.4%	74.5%	81.6%	83.8%	73.4%
**Building vs Natural Scenes**	79.4%	83.9%	75.6%	86.2%	75.7%
**Building vs Fruit**	81.5%	85.4%	78.5%	89.7%	73.6%
**Natural Scenes vs Fruit**	81.1%	74.4%	85.97%	88.8%	74.2%

**Fig 5:**
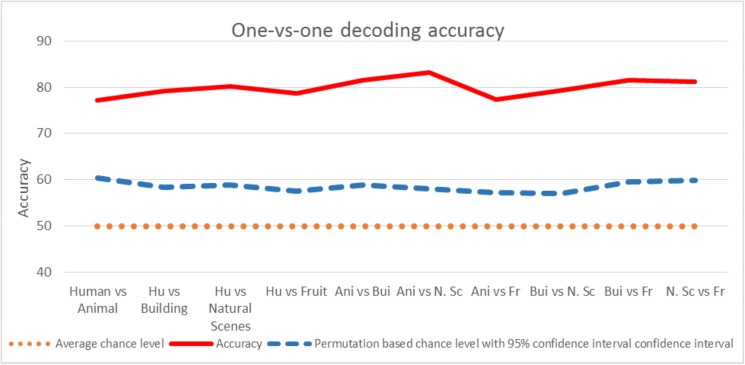
One-vs-one decoding accuracy among all participants for different conditions. Average chance level and permutation based chance level (upper boundary of a 95% confidence interval for chance based on a permutation test) are shown along with the accuracy found using proposed method.

After one-versus-one classification analysis of five categories using proposed method and 128 EEG channels; the same procedure of one-versus-one classification analysis was repeated with only limited significant channels that were found earlier during the baseline task in section 3.1. However, the results for all channels are better than the significant channels and the results for all channels are the only results shown in manuscript. The average accuracy of all participants against the conditions is shown in Fig 5 which is far above than the “pure” chance level (50% chance performance) and “permutation based” chance level which is 57–61% for all conditions after 1000 repetitions. Average accuracy, pure chance level and permutation based chance level is shown in Fig 5 which is almost same as discussed in the study [68]. According to this study for p-value *p<0*.*05* and with approximately 100 samples, the accuracy between pair wise classification should be around 58%.

### Comparison of existing methods with proposed method

**Fig 6:**
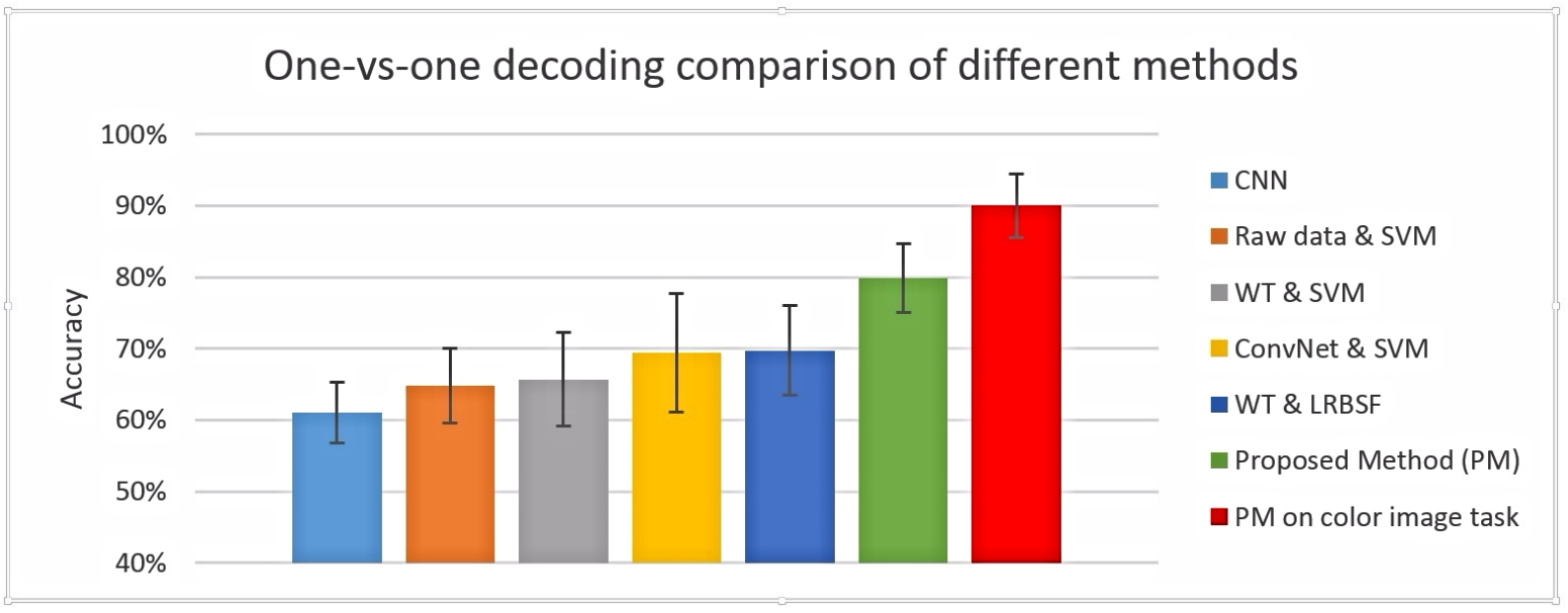
One-vs-one decoding accuracy of different methods including the proposed method for grey scale images and response based colored images.

The proposed method was compared with raw EEG data and other combinations. Initially, we took features based on ConvNet and used an SVM classifier. Second, we used raw EEG data and performed prediction using LRBSF. The complete ConvNet model (LeNet) was also used to find the prediction accuracy. In this way, raw data was given to the ConvNet model and final prediction accuracy was found. It also showed lower accuracy because all current models of deep learning are designed with engineering goals and are not suitable to model brain computations [43]. Finally, in the proposed method, features were extracted using ConvNet, selected using a t-test, and predicted using LRBSF. The proposed method was also applied to another EEG data set, which is a response based task with colored images and has shown very good accuracy (90%) against this task. The comparison of all methods is shown in Fig 6. All these comparisons are based on one-versus-one (pairwise) classification results. Last two higher accuracies are with proposed method (PM). The 79.9% results are for grey level non response based task while 90% results are for response based color image task.

For EEG signals, there are many time and frequency based methods for the extraction of features; however the most popular and reliable way to extract the features is with a wavelet transform (WT), which has information in both the time and frequency domains [24]. In the wavelet transform, the data are decomposed in different frequency components by using scaling and shifting, which give coefficients for the signals. Similarly, the most popular method for classification/prediction is SVM, because it is used in most of the decoding studies [6, 11, 14, 24, 69].

The proposed algorithm is compared with complete ConvNet model, a combination of raw data & SVM, WT & SVM, ConvNet & SVM and finally a combination of WT & LRBSF. In this study, a modification in ConvNet was done; that is why the proposed algorithm is compared with the complete ConvNet model. Similarly, WT and SVM are the most popular methods in EEG data analysis as discussed above, so their combination is also compared with the proposed algorithm. Because we have extracted the features from raw data, a comparison between the proposed method and combination of raw data & SVM was also done. The combination of WT and SVM produced an average accuracy of 65%. Moreover, WT was also used with LRBSF and ConvNet with SVM. In both cases the average accuracy was around 70%, which is less than the complete proposed algorithm but better than all previous comparisons. This shows that just half of the proposed algorithm is better than the most popular current methods (WT & SVM).

**Fig 7:**
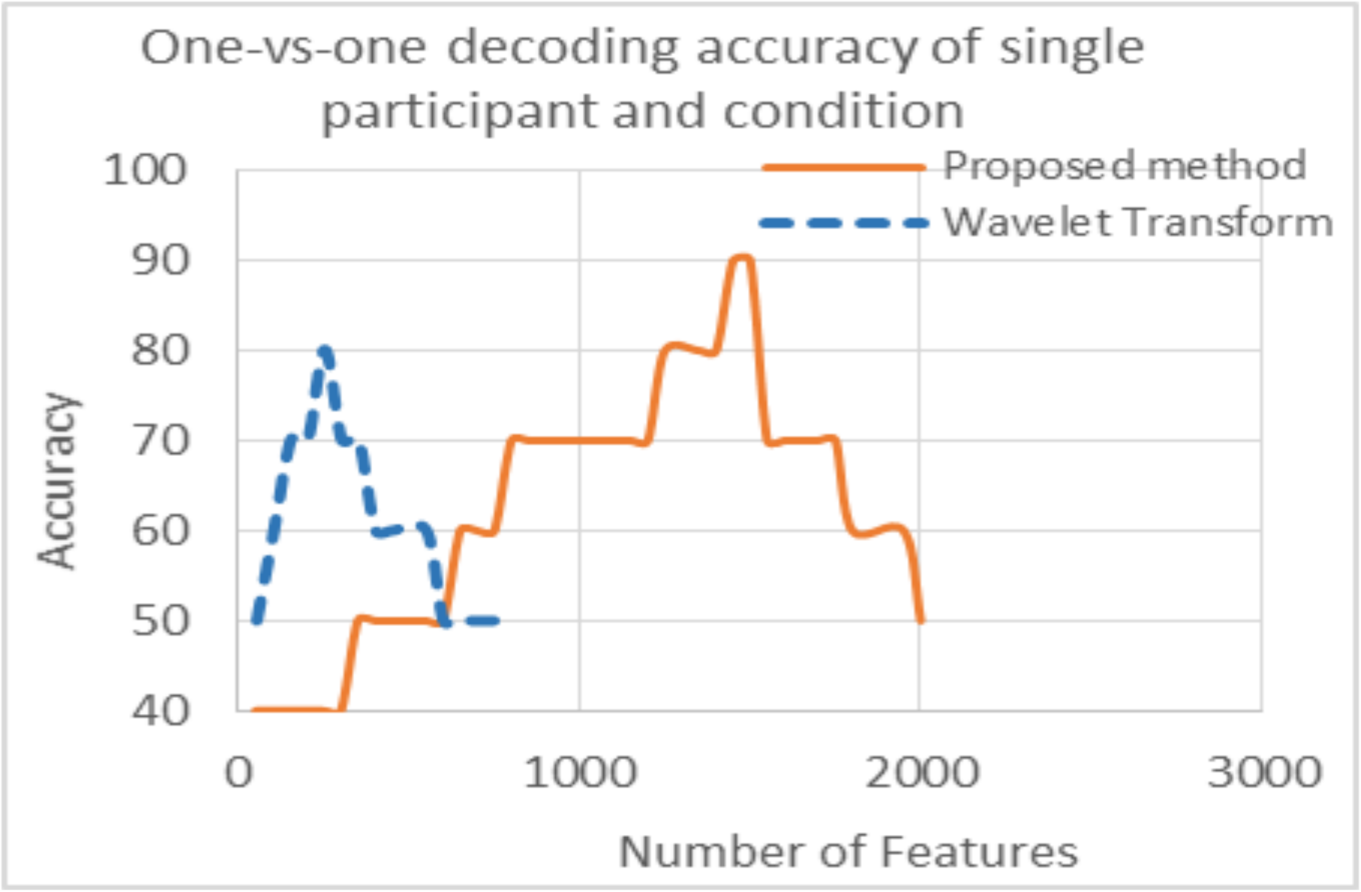
Graph of the decoding accuracy with the number of features for single participant and condition. Dotted line shows results for wavelet transform while solid line for proposed method.

**Fig 8:**
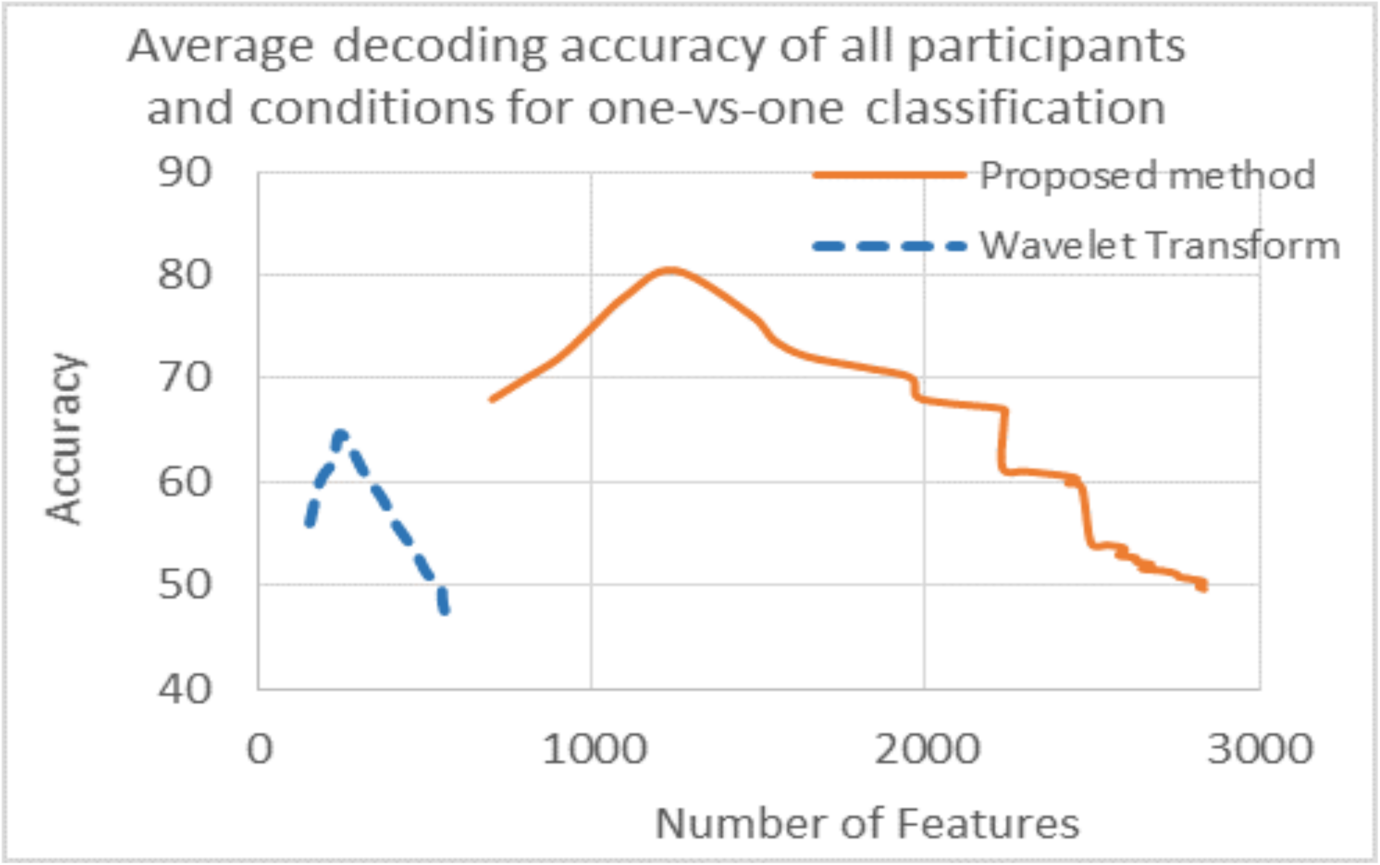
Graph of average decoding accuracy with the number of features for all participants and conditions. Dotted line shows results for wavelet transform while solid line for proposed method.

As discussed earlier, the accuracy was found with a different number of features for all results. The number of features for maximum accuracy in the proposed and existing methods is also different. This is because initially, the number of features were increased by using ConvNet which increased the data dimension. Initially there were 250 samples in 1 sec data means there were 250 features per image (appeared for one sec on the screen)for raw data and 282 for WT while ConvNet had 1100 features after applying different random kernels at convolutional layer which gave same number of feature maps. Due to large number of feature maps, the dimension of the data was increased in ConvNet. The accuracies are plotted against the number of voxels for the proposed method and WT only, as there is a smaller difference in number of features between WT and raw data (Figs 7 and 8).

Fig 7 shows the decoding accuracy with the number of features for a single participant and condition while Fig 8 is the average decoding accuracy with the number of features for all participants and conditions. The pattern is the same in both figures; the blue line shows the number of features vs accuracy for WT and the red line shows the same information for the proposed method. The accuracy difference between WT and the proposed method is shown along the y-axis which has already been discussed; however this difference is also seen along the x-axis in both figures. This is due to the greater number of features used in the hybrid (proposed) algorithm compared to WT and can be seen in Figs 7 and 8. The results clearly show that by enhancing the features using ConvNet, the accuracy is increased significantly. As discussed earlier, ConvNet works by enhancing the original features many times (WT and raw data in this case) which gives better results. This is the main purpose of using ConvNet [27]. In both Figs 7 and 8, results with accuracy of greater than 50% are shown.

To check whether the accuracy differences were statistically different or not, classification accuracies of all conditions were used in a standard paired t-test between the proposed method and all others. Significant p values were found between the proposed and other methods for each comparison. In every case, a significant p value of p < 0.0001 was observed. ANOVA was also applied between the results of all methods simultaneously, which revealed significant results with a p-value of p < 0.00001. In both types of statistical tests, classification accuracy of all categories was used. The significant difference of accuracies between other methods and the proposed one is mentioned in Table 2.

**Table 2:** Significant difference (p-value) of accuracies between the proposed and other methods.

Raw data & SVM vs Proposed Method	WT & SVM vs Proposed Method	ConvNet features & SVM vs Proposed Method	Raw data & LRBSF vs Proposed Method	ANOVA (between the results of all methods simultaneously)
8.34695E-10	1.49134E-10	1.32536E-08	1.08466E-09	4.99E-28

### Multi-class decoding results using proposed method

**Fig 9:**
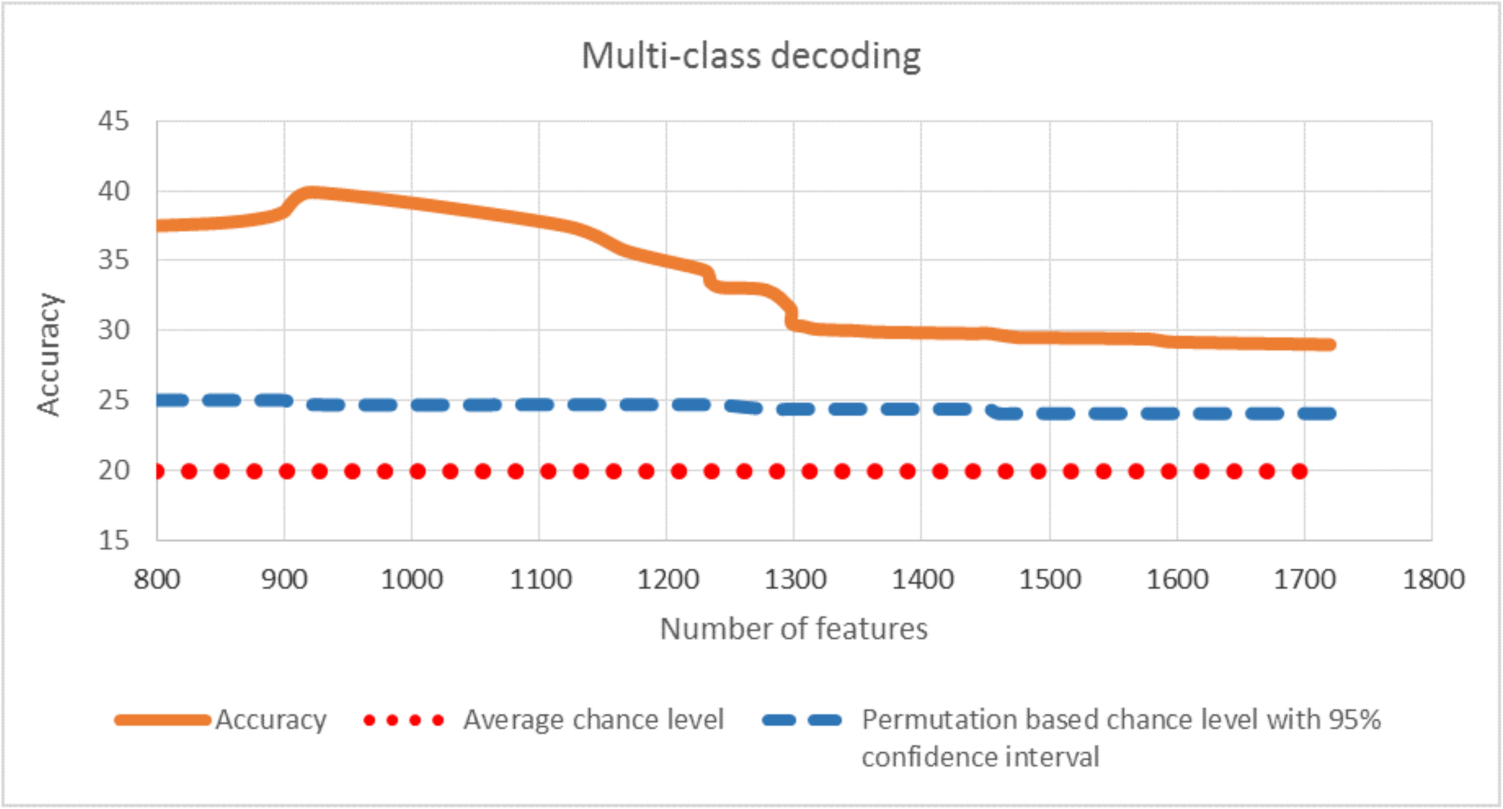
Multiclass decoding accuracy is shown averaged among all participants with respect to number of features. The red dotted line shows the pure chance level while blue line shows the permutation based chance level (upper boundary of a 95% confidence interval for chance based on a permutation test).

**Fig 10:**
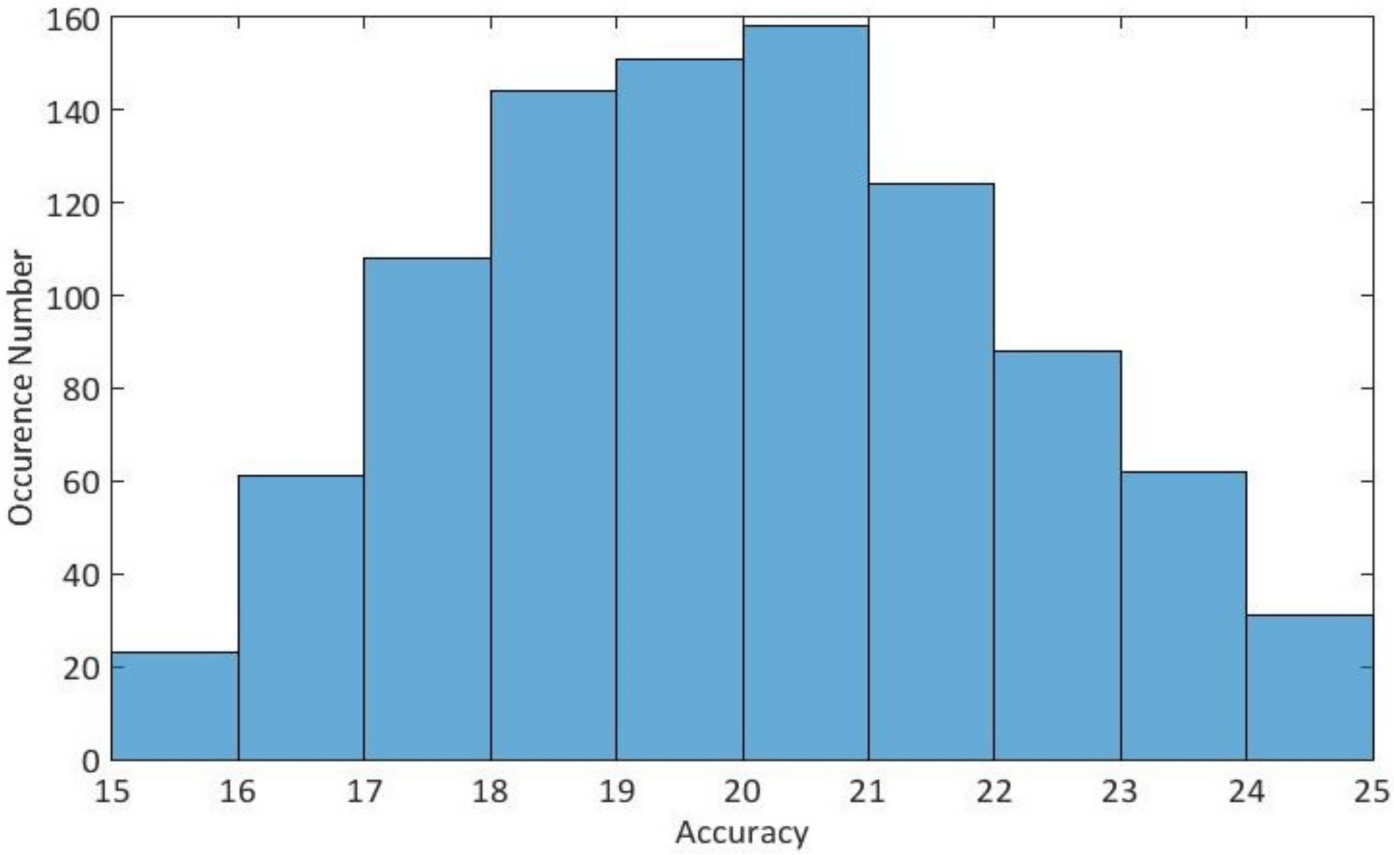
Multiclass accuracy distribution of the permutation based estimation of the chance level for decoding.

For multi-class decoding, LRBSF was applied to all five classes simultaneously. The average result among all participants is 40% which is far above than the “pure” chance level (20% chance performance) and “permutation based” chance level which is 25% after 1000 repetitions. In Fig 9, the accuracy is shown for different numbers of features along with the pure and permutation based chance levels, while the accuracy distribution of the permutation based estimation of the chance level is shown in Fig 10.

## Discussion

The current study investigated the activity of the brain against different categories of stimuli using EEG recordings. A novel algorithm was developed to decode human brain activity with higher accuracy. This algorithm is a constructive addition in EEG data analysis, because accuracy has always been a primary issue in EEG. The proposed algorithm is a complete framework based on MVPA and is the combination of new and existing techniques. It consists of comprehensive data arrangement, a modification of the ConvNet model, a t-test and LRBSF, which predicts the novel data of different object categories with an average prediction accuracy of 79.9%. The novelty in the proposed algorithm for decoding using EEG data is the use of new techniques, which includes modification in ConvNet model, the introduction of LRBSF in EEG data analysis and the processing of EEG data in a new way so that every image is directly part of the final analysis.

In recent years, ConvNet became popular due to its capability of automatic feature extraction and best results compared to other machine learning methods. ConvNet architecture has different layers which brings the data into deeper and detailed form and extracts more significant features using different feature maps. In ConvNet, the connectivity pattern between the neurons is inspired by the organization of the animal visual cortex. In visual cortex, the response to stimuli is in a restricted region which is known as receptive field. There is an overlapping between the receptive fields which covers the visual field. In ConvNet, the response of neurons against the stimuli in the receptive field can be found approximately using convolution operation. The convolutional network is inspired by biological processes and need minimal amount of pre-processing with wide applications especially in video and image recognition. In image processing, the dimension of data is quite high so existing ConvNet architectures have many layers (normally 15–20); more deep layers give better accuracy. In neuroscience, due to lack of existing models [43] some studies have done modifications in existing models for better results [70, 71]. At convolutional layer, different kernels can be defined which give same number of features maps. In this way more and detailed information can be extracted with new features which gives better accuracy, however due to many kernels, feature maps and layers, the dimension of the data is increased. This needs a lot of computation especially for new ConvNet models which require extra hardware for processing. In this study, ConvNet model is modified with limited number of layers and no extra hardware is used to run the algorithm even with more feature maps.

The aim of using LRBSF in parallel to SVM is to see its performance for neuroscience applications as in biometric system, this method has already been proved better than SVM [46, 72]. In LRBSF, KDE is used for the estimation of densities and LRT to do decision about the class. The primary benefit of using KDE is that it is a non-parametric estimator and have no fixed structure, that’s why it depend upon all the data points to reach an estimate. It can also deal the non-Gaussian data in a better way and a flexible way to estimate the densities; moreover it is not very sensitive to the shape of the kernel [73]. Although, most of the models assume that the neuroimaging data (EEG/fMRI) is Gaussian like general linear model; this assumption is mostly not true. The dealing with non-Gaussian data is an advantage of KDE and may be the reason of better performance compared to SVM. This method does not need the optimization of parameters like C and gamma in SVM; in this way the validation data can also be saved. In short, KDE is a non-parametric and non-Gaussian method in which instead of normal distribution we are estimating the densities by finding the density histogram. Moreover kernel estimator smooth out the contribution of each observed data point over a local neighborhood of that data point.

Unlike conventional EEG studies, in this study the data is arranged in a different way. Mostly in EEG, ERP analysis or averaging of data among categories is done, however in this study we have found significant features against every image separately so that every image becomes the part of final analysis. In short, in this study the EEG data is arranged in a better way along with the modified ConvNet architecture and LRBSF. Since both ConvNet and LRBSF are considered as best methods in other areas and are not popular in neuroscience especially LRBSF which has never been used for EEG data before. The proposed algorithm which consists of ConvNet and LRBSF proved that it is a better addition in neuroscience. The main outcome of this study is the decoding analysis of five different categories. These categories are human, animal, building, natural scenes and fruits. This experiment consists of an event related design in which trial order is randomized, which is more accurate and consumes less time because images appeared only for one second with the same duration of rest period. Images of all five categories are presented randomly. It is always a challenging task to differentiate between categories in EEG data analysis; however EEG is commonly used to differentiate the brain states of patients’ vs normal participants or during any task and baseline. There are some studies in EEG which decode brain activity for different types of categories means same type of images but belongs to different groups like animal and humans. In these studies, accuracy is quite low [23, 24, 26] because EEG is mostly used for ERP analysis or for simple tasks in which any task is simply compared with the baseline (eyes open). In a recent study, Crouzet, et al. [26] used MVPA and EEG data for one to one decoding between four categories of taste: salty, sweet, bitter and sour. These researchers achieved decoding accuracy between all categories of 61–65%.

Another finding of this study is the decoding accuracy of the WT and SVM combination during task and baseline, which is 96%. The reason for lower accuracy in the case of five categories is the smaller differences in brain activity between images of different tasks. On the other hand, the difference in brain activity between task and baseline is quite noticeable. The above discussed accuracy difference shows that it is difficult for simple methods like WT and SVM to identify significant differences between the categories. However, the proposed algorithm has extracted detailed information and did prediction effectively. In Figs 7 and 8, it is shown that the proposed algorithm used more significant features compared to the WT which improved the results significantly. In single participant analysis, the WT showed maximum accuracy of 80% with 250 features while the proposed method used 1450 features for an accuracy of 90%. In the average result from all participants, WT used approximately 250 features for an average accuracy of 65% while the proposed method used approximately 1200 features for an average accuracy of 80%.

In another study, Taghizadeh-Sarabi et al. [24] decoded basic objects using EEG and achieved an average accuracy of 70%. In this experiment, color images were shown and the participants had to respond regarding the right and wrong category. The images of one class appeared simultaneously with non-target images. We had also implemented this method on our data and achieved an average accuracy of 71% which was almost same as mentioned in this study. Although we had an advantage of 128 channels data but on the other hand our experiment design was non response based and had grey scale photos. The mentioned study was response based and had color photos (images). To extend our work and to check the exact performance of our proposed algorithm on a response based task with color photos, we redesigned the same experiment and collected EEG data from 12 more participants. The colored images of every class were taken from internet. Unexpectedly, an average decoding accuracy of 90% (Fig 6) was achieved, which is far better than the above mentioned study. In addition to the use of the proposed algorithm, one more factor might have helped in achieving good results. This additional factor is EEG equipment, considering that we have recorded EEG data with a 128 channel EGI system while the previously mentioned study used only 19 channel equipment.

## Conclusion

The present study developed a novel algorithm for discrimination of brain states using noninvasive EEG. The experimental results demonstrated that by using this algorithm the decoding accuracy is increased significantly and shows better percentage accuracy compared to other methods. A combination of the WT and SVM produced good results during task and baseline, however their performance decreased among different categories. In short, the promising results show that the proposed algorithm has the ability to extract more significant information and can predict brain activity efficiently. Secondly, the proposed method worked best with 128 channel equipment and produced an average decoding accuracy of 90% for colored images compared to 70% in the previous study. We conclude that the proposed algorithm has outperformed all the most popular existing methods for brain decoding. To further improve the results, an extension can be done in ConvNet by changing the number and size of filters and pooling layers.

## Supporting information

S1 Data This is the data set of first subject. (EDF)

S2 Data This is the data set of second subject. (EDF)

S3 Data This is the data set of third subject. (EDF)

S4 Data This is the data set of fourth subject. (EDF)
